# Microvesicles: What is the Role in Multiple Sclerosis?

**DOI:** 10.3389/fneur.2015.00111

**Published:** 2015-05-26

**Authors:** Tiziana Carandini, Federico Colombo, Annamaria Finardi, Giacomo Casella, Livia Garzetti, Claudia Verderio, Roberto Furlan

**Affiliations:** ^1^Division of Neuroscience, Institute of Experimental Neurology, San Raffaele Scientific Institute, Milan, Italy; ^2^CNR Institute of Neuroscience, Milan, Italy; ^3^IRCCS Humanitas, Rozzano, Italy

**Keywords:** microvesicles, multiple sclerosis, exosomes, ectosomes, horizontal communication, biomarkers, microglia

## Abstract

Microvesicles are a recently described way of cell communication that has been implicated in a number of biological processes, including neuroinflammation. Widely investigated as biomarkers in oncology and neurological disorders, little is known of the role of microvesicles in the pathogenesis of diseases such as multiple sclerosis (MS). Several evidences suggest that pro-inflammatory microglia and infiltrating macrophages release microvesicles that spread inflammatory signals and alter neuronal functions. We review here available information on microvesicles, with a special focus on microglia and macrophage microvesicles, in the pathogenesis of MS, and as potential biomarkers and therapeutic targets.

## Introduction

Since its first steps neurobiology focused the most part of its efforts on trying to elucidate in great detail the physiology of neurons with very few attention about the other cell types (as a whole referred as glia) because considered as important as just a glue for the neuronal networks assembly and stabilization (1).

Growing attention has been gradually given to glial cells since the demonstration of the multiple roles they have, not only in the maintenance of the brain environment but also in crucial steps of the synaptic transmission: oligodendrocytes and Schwann cells sustain the saltatory conductance of the electric stimuli by insulating specific regions of the axonal tracts, astrocytes provide neurons with some already metabolized neurotransmitters and together with microglia participate in information processing at the level of single synapses or neuronal networks (2). The contribution of microglia to neuronal activity was initially suggested by the observation that multiple contacts occur between microglial cells and neurons at the synaptic terminals (3). In fact, in the developing and adult nervous system, microglia, owing to its phagocytic activity, can physically remodel synapses in a neuronal activity-dependent manner by eliminating excessive or unused contacts (synapse pruning) (4), leading to the formation and consolidation of rearranged synapses driven by sensory experience (synapse maturation) (3, 5–8). In hippocampal neuron cultures, microglia can sustain long-term potentiation (LTP) (9, 10), an observation supported *in vivo* by significant learning and memory deficits in microglia-depleted mice (11). In pathological brain conditions also the basal glutamatergic and GABAergic transmission can be regulated by microglia cells as a consequence of the stimulatory effects of damaged cells-derived ATP on their secretion (12–15); in fact, brain-derived neurotrophic factor (BDNF) secreted from ATP-stimulated cells can tune both excitatory and inhibitory neuronal circuits activity and also support neuronal survival during inflammation (16). In addition, extracellular ATP strongly induces the generation of microvesicles by plasma membrane shedding from responsive cells (17). In this complex picture, microvesicles released by microglia have been shown to cause an excitatory-inhibitory unbalance. They stimulate spontaneous and evoked glutamate transmission in excitatory neurons by facilitating presynaptic release probability (18), while decrease spontaneous GABAergic tone (19). Potentiation of excitatory transmission seems to reside in the capability of microvesicles to interact with neurons and modulate the levels of sphyngosine, which has been found to have a strong impact on neuronal firing activity (20–22), by acting on the lipid metabolizing enzyme acid sphingomyelinase (aSMase). Reduction of GABAergic transmission is instead mediated by endocannabinoids, which are highly enriched in microvesicles, through the activation of presynaptic CB_1_ receptors (19). Here, we will review current knowledge on myeloid cells and their release of microvesicles in neuroinflammatory disorders such as multiple sclerosis (MS).

## Myeloid Cells in MS

Myeloid cells, encompassing microglia, monocytes-derived macrophages and resident-CNS macrophages, play an important role in the pathogenesis of MS and its animal model experimental autoimmune encephalomyelitis (EAE). MS and EAE, in fact, are characterized by the rapid recruitment of blood-borne monocytes, the reaction of resident microglia and perivascular macrophages, along with the recruitment of T cells (23). Many studies have demonstrated that reactive microglia and macrophages can be found in white matter lesions (early and late) and in gray matter subpial lesions (24). Macrophages within CNS lesion sites are difficult to distinguish from reactive microglia, since they both are amoeboid-shaped and express the same antigenic markers. Many authors refer to these cells collectively as macrophages/microglia or as mononuclear phagocytes. The importance of these cells in the MS pathogenesis is demonstrated by several EAE studies: a marked reduction in disease severity is observed when reactive microglia/monocytes are killed either by ganciclovir administration to EAE induced in CD11b-HSV-TK mice (25), or using clodronate liposomes (26). Moreover, inflammatory monocytes (CCR2^+^ and/or Ly-6C high) have been shown to promote EAE progression, while CCR2-deficient mice are resistant to EAE (24, 27, 28). The monitoring of microglial reaction *in vivo* was made possible by the discovery of the radiolabeled molecule 11C(R)-PK11195177, a ligand for the benzodiazepine receptor whose expression in the CNS is increased in reactive microglia (29). A recent study showed correlation between clinical disability and PK11195 PET binding in the cortex of MS patients (30). Both MS and EAE are characterized by a dramatic increase in bound radiolabel in both inflamed and normal appearing white matter on MRI. The latter increase in 11C(R)-PK11195 binding potentially indicates subtle microglial reaction, supporting the hypothesis that microglia reaction underlies early tissue damage preceding demyelination and lesion formation (31). Microglia/macrophages have many different functions and can act in either a beneficial or detrimental fashion in MS pathogenesis. First of all, mononuclear phagocytes are involved in demyelination and phagocytosis of the degraded myelin (32). Inflammation in MS leads to a massive entry of blood-derived macrophages into brain parenchyma. These cells transform into foamy macrophages in the presence of myelin debris and interact with invading T cells (23). At the same time, local inflammatory stimuli lead to a rapid reaction of brain resident microglia and macrophages, which transform into phagocytic cells in the presence of debris. Morphological transformation of myeloid cells also works in reverse: macrophages freshly recruited from the blood stream to the CNS may adapt to the neural environment and undergo remarkable structural remodeling, gradually developing branched processes and transforming into microglia-like ramified cells. Thus, both populations – resident microglia and hematogenous macrophages – contribute to the phagocytic removal of myelin and oligodendrocytes (23). Mononuclear phagocytes are found in most – if not all – MS lesions, and finding myelin degradation products engulfed within tissue macrophages/microglia remains one of the most reliable histological markers of active demyelination (33). Phagocytic activity by macrophages and microglia in MS can be seen as a double-edged sword; on the one hand, it is beneficial by clearing cellular debris, but on other the hand, it is destructive for CNS tissues (34, 35). In addition, microglial/macrophage cells contribute to MS and EAE pathogenesis through antigen presentation, expressing MHC class II and co-stimulatory molecules (CD83/CD40) (23, 36). Microglia express all known TLRs (TLR 1–13) and these receptors are pivotal for the generation of neuro-immune responses (37–40). Microglia/macrophages also promote inflammation and tissue damage (i) by secretion of pro-inflammatory cytokines, reactive oxygen intermediates, and proteinases, (ii) by release of soluble factors that are chemotactic and activate other lymphocytes, and (iii) by physically disrupting the local extracellular environment, thereby facilitating leukocyte influx into the CNS and leading to tissue damage (41). Microglia/macrophages can act as antigen presenting cells and therefore re-prime or reactivate T cells in lesion sites (34, 42). Although the above-mentioned studies emphasize the negative contribution of microglial/macrophage cells in MS or EAE pathology, there is evidence indicating a protective function of these cells in EAE and MS. Indeed, mononuclear phagocytes can inhibit the adaptive immune responses in the CNS, by secreting anti-inflammatory cytokines (IL-10 and TGFβ) or by expressing inhibitory molecules such as PD-L1 (B7-h1) (43). Triggering receptor expressed on myeloid cells-2 (TREM2), a specific membrane-bound receptor involved in reducing inflammation and promoting phagocytosis, is increased in the CSF of both progressive and relapsing–remitting MS patients (24, 41, 44). Microglia/macrophages are also capable of secreting neurotrophic factors such as BDNF, insulin-like growth factor-1 (IGF-1), and neurotrophin 3 (NT3) and thus may contribute in promoting neural survival and neurogenesis (45, 46), although inducing the release of NO by astrocytes (47). Mononuclear phagocytes have been shown to have a beneficial role in EAE, as remyelination was impaired after depletion of macrophages with clodronate liposomes (48). However, the relevance of these findings to human demyelinating diseases is still unclear. Thus, in MS, microglial cells and macrophages may display both neurodestructive and neuroprotective functions (35). Switching their function from neurodestructive to neuroprotective may be beneficial in preventing chronic demyelination and axonal loss and thus preventing disease progression.

## Microvesicles: Novel Biomarkers of CNS Diseases

**Figure 1:**
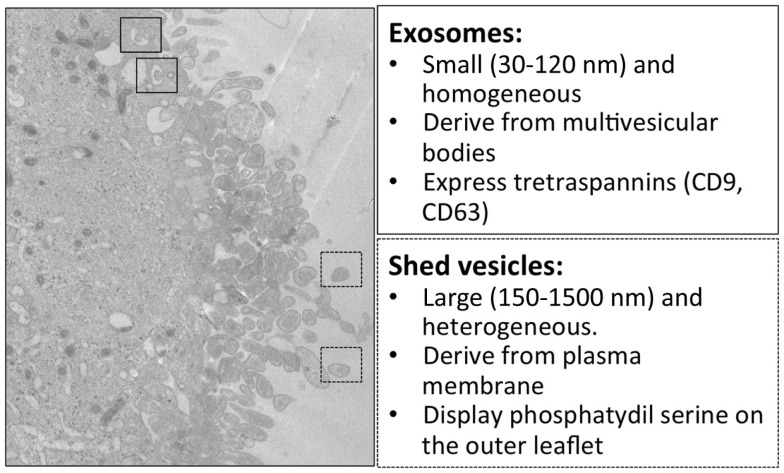
**Electron microscopy and main features of microglial exosomes and shed vesicles**. Transmission electron microscopy of the human CHME-5 microglial cell line exposed to ATP (500 μM); massive blebbing of the membrane occurs in a short time (5–7 min). In this image, multivesicular bodies containing exosomes are indicated in the solid squares, while released shed vesicles are indicated in dashed squares. Corresponding features are reported in the boxes on the right.

**Figure 2:**
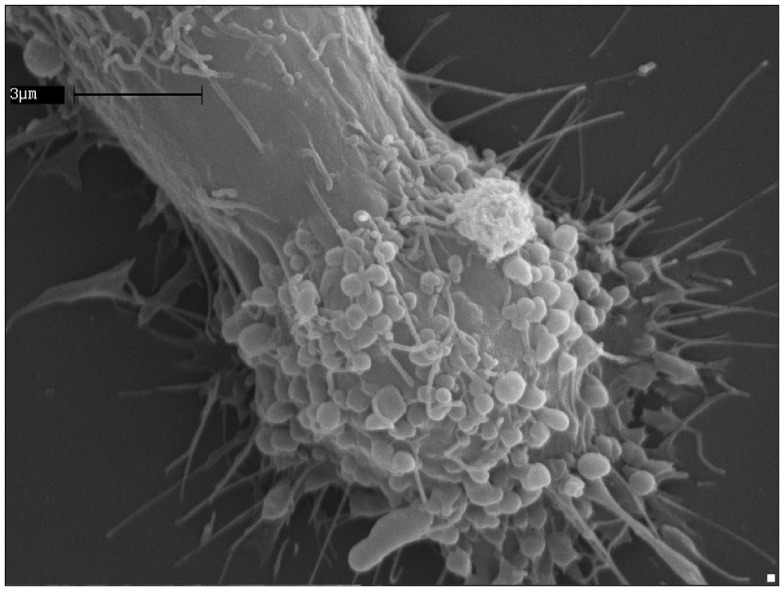
**ATP induces extensive blebbing and shedding of myeloid-cell plasma membrane**. A human microglia cell of the CHME-5 line exposed to ATP (500 μM); the massive blebbing of the membrane occurs in a short time (5–7 min), witnessing the strength of the connections between the purinergic signaling receptors activation and the cell surface dynamics.

In multicellular organisms, communication between cells is a fundamental process to guarantee adequate coordination among different cell types within tissues and to exchange information. Classical means of cell communication are represented by three main mechanisms: (i) cell-to-cell contact-dependent signaling, mediated by adhesion molecules and gap junctions; (ii) secretion and diffusion of signaling molecules that can act on a short distance target (paracrine signaling) or on a longer one (endocrine signaling); (iii) synaptic signaling (typical of neurons) in which neurons, through their axons, can reach distant target cells and create with them a junction called “chemical synapse.” In addition to these described processes, other mechanisms of cell communication have recently attracted increasing interest: tunneling nanotubes (49) and extracellular vesicles (EVs). Here, we focus on EVs. EVs are spherical membrane vesicles heterogeneous in size (up to 1 μm in diameter) and limited by a lipid bilayer containing hydrophilic soluble components. EVs can form either at the plasma membrane or in the lumen of internal compartments and are secreted into the extracellular space. Irrespective of their origin, these vesicles contain cytosol and have the same membrane topology of parental cells, exposing at their outer surface the extracellular side of the bilayer of donor cells. Because their membrane orientation is the same as that of the donor cell, they can be considered to be miniature versions of the donor cell (50). EVs are thought to function as shuttles for the delivery of cargo between different cells within an organism (51). Indeed, EVs carry receptors, bioactive lipids, proteins, and, most importantly, nucleic acids, such as RNA and microRNA (miRNA); thus, EVs may modify the phenotype and functions of target cells (52). Nowadays, three types of EVs are distinguished unanimously: exosomes, microvesicles (MVs, also called shedding vesicles, ectosomes, shedding MVs, or microparticles), and apoptotic bodies, also called apoptotic blebs or apoptotic vesicles (50, 53, 54) (Figure 1). Exosomes are secreted membrane vesicles (approximately 30–120 nm in diameter) formed intracellularly and released from exocytosis of multivesicular bodies (55, 56), whereas apoptotic bodies (approximately 500–4000 nm in diameter) are released by dying/apoptotic cells (57) (Figure 1). MVs are heterogeneous membrane vesicles (approximately 200–1500 nm in diameter), which bud directly from the plasma membrane (58) (Figure 1). All these different types of vesicles are present simultaneously in the extracellular environment of tissues (Figure 2). We here focus on MVs. Upon vesciculation, released MVs can both remain in the extracellular space in close proximity to the cell of origin or diffuse in biological fluids (59). MVs mediate cell-to-cell communication interaction with target cells by different mechanism: (a) stimulation of target cells by acting as signal complex, (b) transfer of surface receptors from one cell to another, (c) delivery of proteins, mRNA, and miRNA, (d) vehicle mechanism to transfer infectious particles (e.g., HIV, prions) (60). Growing evidence indicates that MVs contribute to the pathogenesis of cancer, inflammation, autoimmune, and cardiovascular disease (61). Numbers of MVs in biological fluids seem to correlate with the active phase of many diseases, thus MVs are currently under investigation as possible biomarkers.

## Microvesicles in Multiple Sclerosis

Several studies demonstrate that EVs (both MVs and exosomes) play an active role during the pathogenesis of MS and EAE. MVs from the brain endothelium have been shown to activate both CD4^+^ and CD8^+^ T cells toward neural antigens in the absence of any other stimulatory signal and may represent the potential initial step of brain autoimmunity (62). Increased numbers of MVs have been reported in the blood and in the CSF of MS patients as compared to healthy controls. MVs have been proposed to play a role in inflammatory progression and lesion repair. Injection of microglial MVs into the brain of mice with subclinical EAE recruits inflammatory cells to the injection site (58). However, aSMase deficient mice, which are impaired in MV production in microglia and astrocytes, are largely protected from EAE, although these genetic mutant mice may have defects also in other compartments relevant to the disease. MVs released from BBB-endothelial cells, platelets, leukocytes, myeloid cells (monocytes/macrophages/microglia), and astrocytes, are involved in the pathogenesis of MS (63). The first step is the migration of inflammatory cells through the BBB. Endothelial MVs carry metalloproteases that promote BBB disruption (64) and molecules inducing endothelial activation (65). Endothelial MVs can interact and form complexes with monocytes and activate them (66). Also, activated T cells release MVs containing the chemokine CCL5 and arachidonic acid, which recruite monocytes and up-regulate ICAM-1 on endothelial cells and LFA1 and Mac-1 on monocytes (63, 67). Platelet-derived MVs express on their surface P-selectin, which binds to PSGL-1 and PECAM-1 from lymphocytes by increasing the expression of integrins such as α4β1 (VLA-4) (63). This process promotes the binding of lymphocytes to the endothelium (68) and their transmigration into the CNS. Moreover, together with endothelial-derived MVs, platelet-derived MVs from MS patients have been shown to increase the permeability of endothelial layers *in vitro*, suggesting their involvement in the disruption of the BBB (69). In the CNS compartment, MVs shed by myeloid cells contain components of the inflammasome, such as IL1-β, MHC-II, and others (70).

Since apparently the level of MVs in biological fluids is associated with the activation of cells involved in MS pathogenesis, several authors have proposed them as plausible biomarkers. The inconsistency of results produced so far depends mainly on pre-analytical errors, technological issues related to MVs measurement, ambiguity in EVs definition (MVs vs. exosomes), correlation with clinical and paraclinical parameters such as disease subtype and severity (EDSS), and MRI.

Concerning studies on CSF, Scolding et al. described, for the first time, the presence of oligodendroglial MVs in the CSF of patients with MS (71). More recently, our group revealed the presence of increased levels of myeloid cells-derived MVs (Ib4^+^) in the CSF of relapsing–remitting MS patients, compared with healthy controls (58). Higher number of CSF MVs was especially associated to acute disease phase, as compared to stable or chronic phases. In fact, MVs counts in the CSF correlate linearly with gad^+^ lesions at MRI. Accordingly, in EAE the concentration of CSF MVs perfectly mirrors the course and severity of both relapsing and chronic EAE peaking at onset and during clinical relapses, and decreasing in the chronic phase of the disease. When we investigated MVs as a possible biomarker in MS, based on ROC analysis, we obtained a sensitivity of 85% and specificity of 100% for distinguishing clinically isolated syndrome (CIS) patients from healthy controls, and a sensitivity of 82% and specificity of 82% for differentiating stable (relapse-free patients) from relapsing MS patients (58). Unfortunately, studies of MVs in the CSF of MS patients are difficult to perform, both because of the scarcity of material usually available, and because patients for ethical concerns can not perform serial lumbar punctures to assess MVs’ trend over time. For these reasons, many studies have focused on the evaluation of MVs’ levels in the peripheral blood, trying to correlate their number with some clinical and instrumental parameters.

CD31^+^ endothelial MVs, identified in plasma samples by FACS, have been associated to clinical and neuroradiological exacerbation of MS, while CD51^+^ endothelial MVs have been found elevated in both relapsing and remitting MS patients as compared to controls (65). The same group has confirmed their findings in 2004, further describing that most endothelial MVs can be detected in the blood in the form of conjugates with other cells, especially monocytes (66), while described that, similarly to stroke, platelet-derived MVs, despite elevated in the plasma of MS patients as compared to controls, display a reduced discriminating power between health and disease (68). Jimenez et al. (72) reported an increase of CD54^+^ and CD62E^+^ endothelial MVs in the plasma of MS patients during relapse compared to remission. Sáenz-Cuesta et al. (73) demonstrated a significant difference also in CD61^+^ (platelet marker), CD45^+^ (lymphocyte marker), and CD14^+^ (monocyte marker) MVs counts in samples from MS patients compared to those from healthy controls. MVs were especially high in relapsing–remitting patients, while secondary progressive MS patients were similar to healthy controls. Plasma MVs levels in this work appear to reflect short-term active inflammation rather than disease severity, as measured by EDSS, or disease duration or patients age (73).

Considering MVs as biomarkers of therapeutic efficacy in MS, Jimenez et al. (72) report that IFN-β 1b reduces the release of endothelial MVs induced by plasma from MS patients. IFN-β 1b also reduces monocyte–endothelial MVs complex formation and transendothelial migration *in vitro* (72). Sheremata et al. (74) report the ability of IFN-beta1a to reduce the number of CD31^+^ endothelial MVs in plasma of relapsing–remitting MS patients as early as three months after treatment initiation without, however, any correlation with MRI activity. Lowery-Nordberg et al. performed a prospective study, measuring changes in plasma of CD31^+^, CD146^+^, and CD54/ICAM-1^+^ endothelial MVs in 16 patients with RR-MS before and after 3, 6, and 12 months of therapy with interferonbeta1a (Rebif44^®^). They found that plasma levels of CD31^+^, and CD54^+^ – and not CD146^+^ – endothelial MVs were significantly reduced by treatment with IFNβ. Moreover, they demonstrated a significant association between the decrease in plasma levels of MVs and the decrease in the number and volume of contrast enhancing T1-weigthed MRI lesions (75). On the contrary, in a recent study measuring plasma platelet MVs, lymphocyte MVs, and monocyte MVs, Sáenz-Cuesta et al. (73) reported, using flow cytometry (probably focusing on MVs), higher counts of all three MVs subtypes in IFN-β and natalizumab-treated patients (73). Dawson et al. demonstrated that fingolimod inhibits aSMase (76), the enzyme that controls MVs production (17). In our work (58), we hypothesized that fingolimod might inhibit myeloid cells-derived MVs shedding from reactive microglia. Indeed, EAE mice treated with fingolimod displayed a reduction of CSF myeloid MVs to baseline levels. Through this mechanism, fingolimod may inhibit the spreading of inflammatory signals throughout the brain parenchima (58).

## Conclusion

There is still incomplete information on the role of microvesicles in MS, but available evidence points to a relevant role, both in spreading pro-inflammatory signals and in altering neuronal functions. The potentially relevant role in the pathogenesis of the disease, underlines how microvesicles, especially those released by microglia/macrophages, may represent precious biomarkers, although for the moment they only can indicate, for example, the presence of microglial reaction, but are not linked to a specific disease. Involvement in pathogenic mechanisms may suggest also microvesicles as possible therapeutic targets. The development of adequate technology for the detection and analysis of microvesicles will provide in the near future the answer to the questions posed in this review and reveal if new and valuable information on MS is indeed enveloped in these microscopic nanoparticles.

## Conflict of Interest Statement

Roberto Furlan and Claudia Verderio share a patent on myeloid microvesicles in neurological disorders. The other co-authors declare that the research was conducted in the absence of any commercial or financial relationships that could be construed as a potential conflict of interest.
